# FTO diversely influences sensitivity of neuroblastoma cells to various chemotherapeutic drugs

**DOI:** 10.3389/fphar.2024.1384141

**Published:** 2024-09-04

**Authors:** Meizhen Lin, Zhongyan Hua, Zhijie Li

**Affiliations:** ^1^ Department of Pediatrics, Shengjing Hospital of China Medical University, Shenyang, China; ^2^ Liaoning Key Laboratory of Research and Application of Animal Models for Environmental and Metabolic Diseases, Medical Research Center, Shengjing Hospital of China Medical University, Shenyang, China

**Keywords:** neuroblastoma, FTO, chemotherapy sensitivity, paclitaxel, etoposide, cisplatin

## Abstract

Chemotherapy resistance is a significant factor in treatment failure in patients with neuroblastoma (NB), and it directly affects patient prognosis. Therefore, identifying novel therapeutic targets to enhance chemosensitivity is essential to improve the cure rate and prognosis of patients with NB. In this study, we investigated the role of FTO in chemosensitivity of NB cells to various chemotherapeutic drugs. Our results showed that high FTO expression was positively correlated with increased survival probability and favorable prognostic factors in patients with NB. FTO overexpression inhibited cell proliferation, whereas FTO knockdown promoted cell proliferation in NB cells. FTO expression alteration had contrasting effects on NB cells’ sensitivity to etoposide but had no significant impact on sensitivity to cisplatin. Downregulation of FTO reduced the sensitivity of NB cells to paclitaxel, whereas upregulation of FTO enhanced its sensitivity. Additionally, the sensitivities between patients with lower and higher FTO expression to various chemotherapeutic drugs or small-molecule inhibitors were different. Thus, FTO affects the sensitivities of NB cells differently depending on the different chemotherapeutic drugs and small-molecule inhibitors. This finding may guide physicians and patients choose the appropriate chemotherapeutic drugs or small-molecule inhibitors for treatment.

## 1 Introduction

Neuroblastoma (NB) is the most common extracranial malignant solid tumor originating from the sympathetic neural crest in children, accounting for 7%–10% of pediatric tumors. The incidence rate of NB is approximately 0.3–5.5 in 100,000 children (Gatta et al., 2014; Maris et al., 2007). According to the International Neuroblastoma Risk Group (INRG), clinical patients can be categorized into low, intermediate, and high-risk groups. The survival rate for low and intermediate-risk patients with NB can reach 90%, that for high-risk patients remains less than 50% (Bender et al., 2023; Campbell et al., 2023; Matthay et al., 2016; Park et al., 2019; Qiu and Matthay, 2022), and that for patients with relapse or resistance to chemotherapy is only 40% (Ponzoni et al., 2022). Chemotherapy is the most commonly used method to treat patients in the clinic. Chemoresistance is a leading cause of treatment failure, highlighting the need to explore new therapeutic targets and strategies for patients with NB (Li et al., 2023).

The fat mass and obesity-associated gene (FTO) is a nuclear protein of the AlkB-related non-heme iron and 2-oxoglutarate-dependent oxygenase superfamily, playing a role in regulating energy metabolism, fat formation, cell differentiation, neurodevelopment, and tumor progression. Particularly, it plays an important role in various types of cancers (Li et al., 2022; Zuidhof and Calkhoven, 2022b). FTO is involved in the development of a variety of tumors, including cell proliferation, chemotherapy sensitivity, apoptosis, and self-renewal, through different regulatory mechanisms (Deng et al., 2018; Relier et al., 2021; Su et al., 2020; Xu et al., 2020; Zuidhof and Calkhoven, 2022a). Consequently, FTO inhibition has significant anticancer effects (Lan et al., 2021; Ma et al., 2019; Wang et al., 2022a; Yankova et al., 2021). FTO contributes to drug resistance in various cancers, including cervical cancer (Zhou et al., 2018), gastric cancer (Feng et al., 2021), melanoma (Yang et al., 2019), breast cancer (Wang et al., 2021), leukemia (Yan et al., 2018), colorectal cancer (Zhao et al., 2021), bladder cancer (Wen et al., 2020), and glioma (Xiao et al., 2020). Extensive research has indicated the critical role of FTO in various tumors. However, to date, no studies have investigated the role of FTO in NB. Our preliminary NB-related RNA-seq revealed significant differences in FTO expression. In this study, we investigated the effects of FTO on the sensitivity of NB cells to chemotherapeutic drugs (etoposide, cisplatin, and paclitaxel). The results showed that high FTO expression correlated with good prognostic factors for patients with NB. Overexpression of FTO inhibited cell proliferation and knockdown of FTO expression promoted NB cells’ proliferation. Changes in FTO expression diversely influenced the sensitivity of NB cells to paclitaxel, etoposide, and cisplatin. Furthermore, the sensitivity of the patients to different chemotherapeutic drugs or small-molecule inhibitors was predicted. Our results suggest that FTO diversely influences the sensitivity of NB cells to different chemotherapeutic drugs and small-molecule inhibitors, which may help clinical patients choose the appropriate treatment.

## 2 Materials and methods

### 2.1 Cell culture

Two human NB cell lines [SK-N-AS (AS) and SK-N-BE2 (BE2)] and a mouse fibroblast cell line (NIH3T3) were used in this study. SK-N-BE2 (BE2) represents the MYCN-amplified cell line, whereas SK-N-AS (AS) represents the MYCN-non-amplified cell line. All cell lines were gifts from Dr. Carol J Thiele (Cellular and Molecular Biology Section, Pediatric Oncology Branch, National Cancer Institute, National Institutes of Health, Bethesda, MD, United States). NB cells were cultured in RPMI-1640 medium (Solarbio, China) containing 10% FBS (Gibco, Israel), antibiotics (a mixture of penicillin and streptomycin; working concentration 100 U/mL) (BI, Israel), and 2 mM glutamine (BI, Israel) at 37°C in a 5% CO_2_ incubator. NB cells in the logarithmic growth phase were used for the experiments.

### 2.2 Cell transfection

Small interfering RNAs (siRNAs) were purchased from Tongyong (Anhui, China). The target sequences of siRNAs are as follows: control siRNA: 5′-UUC​UCC​GAA​CGU​GUC​ACG​UTT-3′; FTO siRNA#1: 5′-CUA​CAA​CGG​ACA​AGA​UGA​ATT-3′; FTO siRNA#2: 5′-CGG​UGG​CAG​UGU​ACA​GUU​ATT-3′; FTO siRNA#3: 5′-GGA​AGA​AGA​UGG​AGG​GUG​UTT-3′. The FTO overexpression plasmids (NM_001080432, pEZ-M39 vector) and empty vector were purchased from GeneCopoeia (United States). AS, BE2, and NIH3T3 cells were seeded into 6-well plates at 1 × 10^5^/well, 5 × 10^5^/well, and 1 × 10^5^/well, respectively, and cell adhesion was maintained overnight. siRNAs or plasmids were transfected into cells using jetPRIME according to the manufacturer’s instructions.

### 2.3 Cell treatment

To explore the function of FTO in response to paclitaxel, etoposide, and cisplatin, 16 h after the transfection, cells were seeded into 96-well plates, and then treated with paclitaxel (AS, 3 nM; BE2, 3 nM), etoposide (AS, 1.5–2 μg/mL; BE2, 2 μg/mL), or cisplatin (AS, 0.5 μg/mL; BE2, 2 μg/mL), for 72 h.

### 2.4 Microarray data and data analysis

The relationship between the expression of FTO and the prognosis of clinical patients with NB was analyzed via the R2 database (https://hgserver1.amc.nl/cgi-bin/r2/main.cgi?species=hs) using a dataset of 498 samples from patients with NB across seven countries. The patients’ ages at diagnosis varied from 0 to 295.5 months (median age, 14.6 months). Tumor stages were classified according to the International Neuroblastoma Staging System (INSS). Events were defined according to the revised International Neuroblastoma Response Criteria. The significance of the relationship was determined using Pearson correlation or one-way ANOVA analysis, with *p* values less than 0.05 considered statistically significant (Zhang et al., 2015).

### 2.5 IncuCyte ZOOM live imaging system and cell survival analysis

After 16 h of transfection, AS, BE2, and NIH3T3 cells were seeded into 96-well plates at densities of 0.5 × 10^4^/well, 1.5 × 10^4^/well, and 0.5 × 10^4^/well, respectively. The cell confluence rate in each well was recorded using the IncuCyte ZOOM live-cell dynamic imaging system. After 72 h of drug treatment, cell proliferation and survival were detected using a Cell Counting Kit-8 (CCK-8 assay) (GlpBio, United States). The CCK-8 assay was performed according to the manufacturer’s instructions. Absorbance was measured at 450 nm.

### 2.6 Colony formation assay

After transfection, AS and BE2 cells were seeded into 6-cm dishes at densities of 3 × 10^3^/well and 5 × 10^3^/well, respectively. After 10 days of observation, the cells grew into visible cell clones. The cells were then fixed with 4% paraformaldehyde for 30 min, stained with Giemsa working solution for 30 min, and carefully washed with ultrapure water. After being air dried, the whole 6-cm dish was photographed. Finally, the number of colonies in each group was counted using ImageJ software.

### 2.7 Western blot

AS and BE2 cells were harvested following transfection with FTO siRNAs (#1, #2, #3) or FTO overexpression plasmid for 48 h. Total protein was extracted using the whole-cell lysis assay kits (Keygen Biotech, China), according to the manufacturer’s protocol. For each sample, 30 µg of protein was loaded onto SDS-PAGE gels, transferred to PVDF membranes, and blocked with 5% non-fat milk to prevent non-specific antibody binding for 2 h at room temperature. The membranes were then incubated overnight at 4°C with specific primary antibodies: FTO antibody (Proteintech, China, diluted at 1:1,000) and β-actin antibody (Proteintech, China, diluted at 1:5,000). Subsequently, the membranes were washed thrice for 5 min each with Tris-buffered saline-Tween 20 (TBST) and incubated with horseradish peroxidase-conjugated goat anti-rabbit or anti-mouse antibodies (Proteintech, China, diluted at 1:5,000) for 2 h at room temperature.

### 2.8 Quantitative real-time PCR

Total RNA was extracted from NB cells, with 1 µg of RNA utilized for cDNA synthesis employing the PrimeScript™ RT reagent Kit with gDNA Eraser (Takara, Japan). RT-qPCR was performed employing SYBR Premix Ex Taq™ II (Takara, Japan) on a 7500 Fast Real-Time PCR System. Relative quantification of gene expression was performed with the 2 (−ΔΔCt) method. Ct values were standardized to β-actin. The primer sequences are shown below:

**Table:** 

Gene name	Forward primer	Reverse primer
β-Actin (human)	AAC​TGG​GAC​GAC​ATG​GAG​AAA	AGG​GAT​AGC​ACA​GCC​TGG​ATA
FTO (human)	GCT​GCT​TAT​TTC​GGG​ACC​TG	AGC​CTG​GAT​TAC​CAA​TGA​GGA

### 2.9 Chemotherapeutic drugs or small-molecule inhibitors sensitivity analysis

Data for this analysis were obtained from 498 NB patient samples from the GSE49710 dataset available in the GEO database. The relationship between the expression of FTO and chemotherapeutic drugs or inhibitor sensitivity was analyzed using R software with the “pRRophetic” package, which was downloaded from Bioconductor. The significance between groups was analyzed using the Wilcoxon rank-sum test, with *p* values less than 0.05 considered statistically significant.

### 2.10 Statistical analysis

Data are expressed as mean ± SD. Comparisons between two groups were carried out using the Student's t test. Statistical analyses were performed with GraphPad Prism software. Statistical significance was set as *p* values less than 0.05.

## 3 Results

### 3.1 High expression of FTO correlated with good prognosis of clinical patients with NB

**FIGURE 1:**
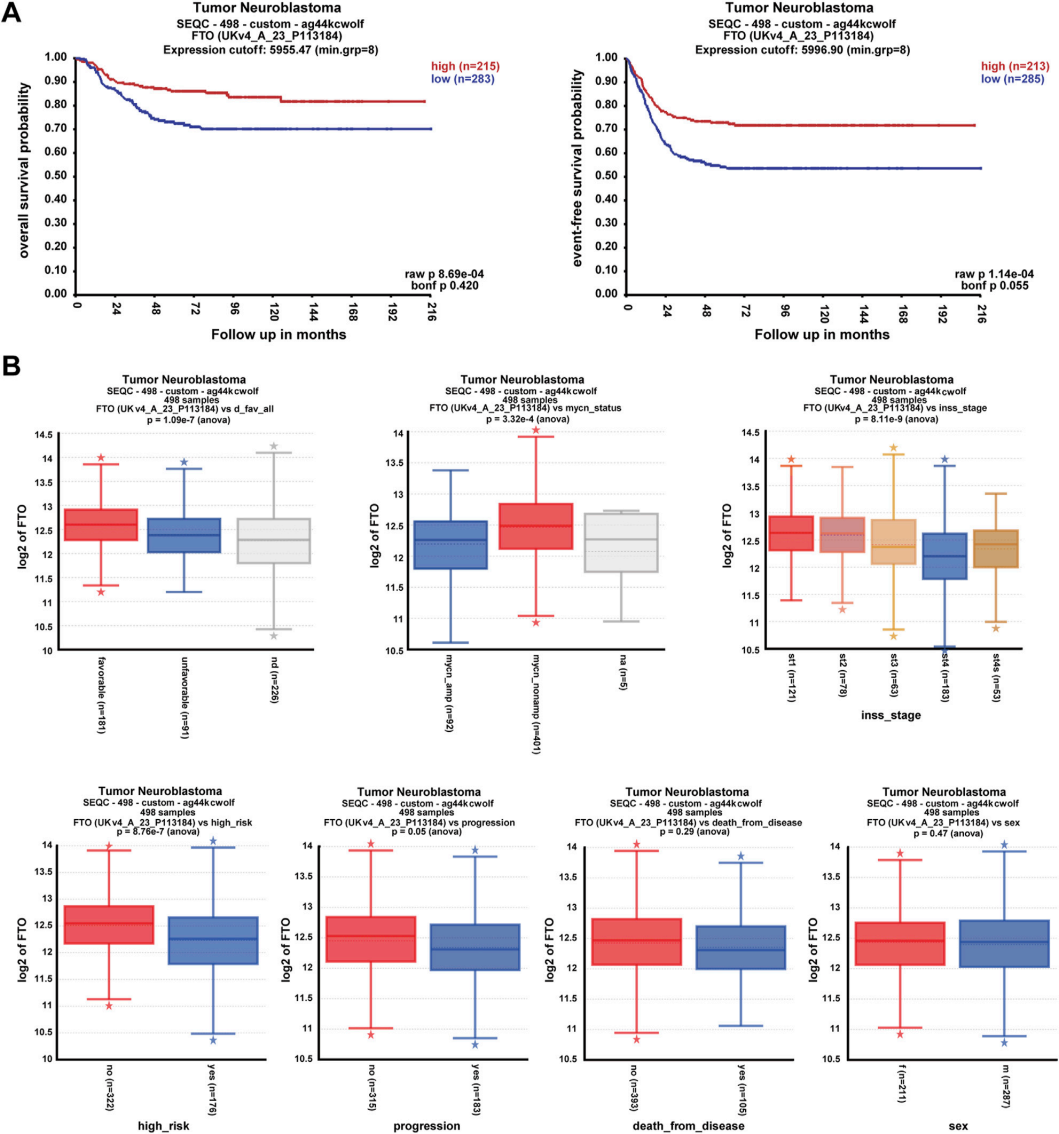
High expression of FTO correlated with good prognosis of clinical patients with NB. **(A)** Correlation of FTO expression with patient survival probability using Pearson correlation. **(B)** Expression of FTO in patients with different clinical pathologic characteristics (favorable or unfavorable histopathology, MYCN, INSS stages, high-risk or no, progression, death from disease, and sex) using one-way ANOVA analysis. * represents out value, normal, or abnormal small observations.

To explore the correlation between FTO expression and the prognosis and clinical pathologic characteristics of patients with NB, datasets comprising 498 patients with NB were selected from the R2 database (https://hgserver1.amc.nl/cgi-bin/r2/main.cgi). Patients with high FTO expression had better overall survival probability and event-free survival probability than patients with low FTO expression (*p* < 0.001) (Figure 1A). Patients with a favorable prognosis, characterized by positive histopathology; non-MYCN amplification; INSS stages 1, 2, 3, and 4S; and no high-risk factors, exhibited significantly higher FTO expression (*p* < 0.001) (Figure 1B). Additionally, patients with no disease progression (*p* = 0.05), no deaths due to the disease (*p* = 0.29), or female sex (*p* = 0.47) demonstrated a trend toward increased FTO expression (Figure 1B). These findings suggest that high FTO expression is associated with better prognosis and favorable clinical characteristics.

### 3.2 Upregulation of FTO inhibited NB cell proliferation

**FIGURE 2:**
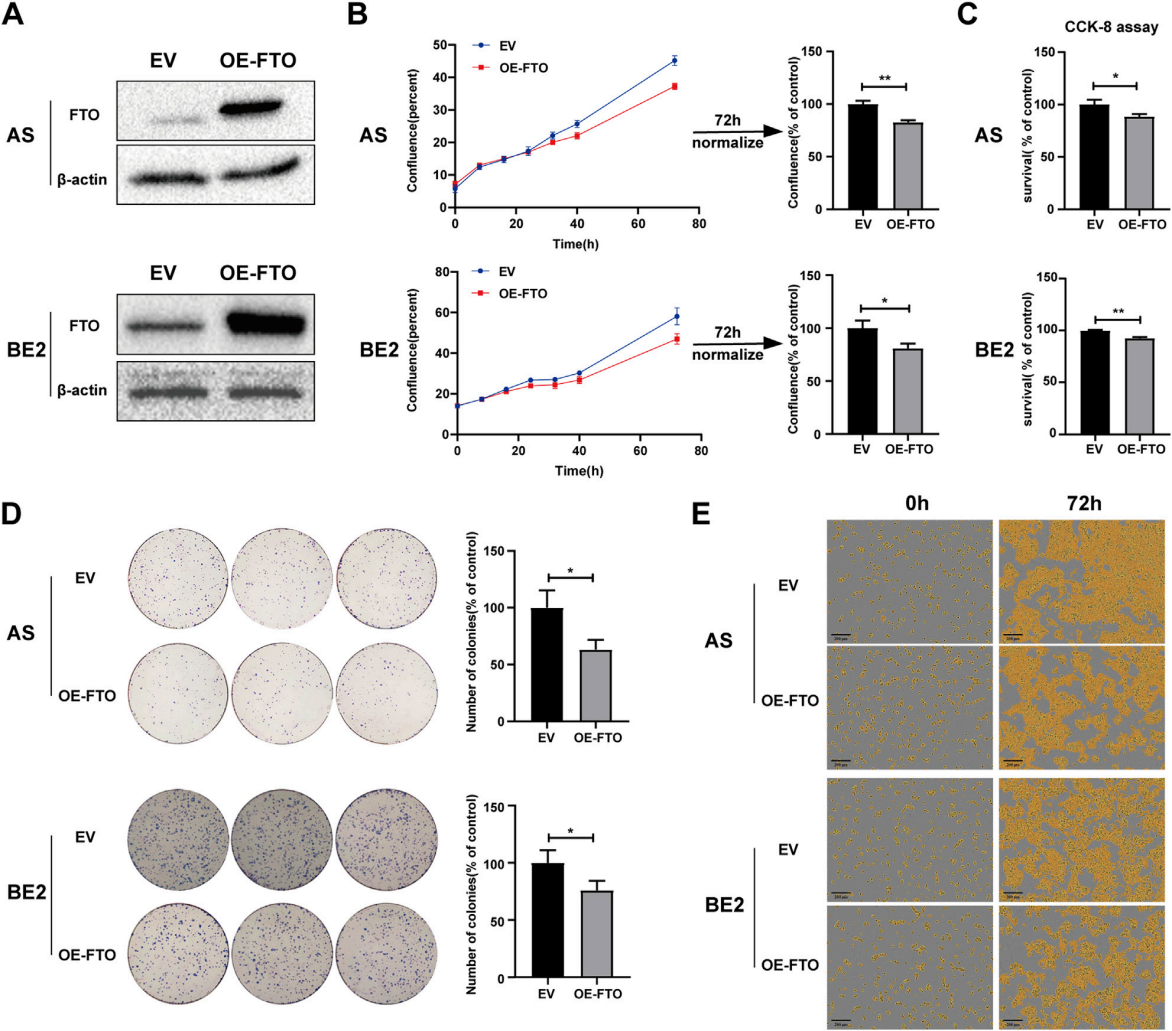
Upregulation of FTO inhibited cell proliferation. **(A)** Expression of FTO in AS and BE2 cells transfected with OE-FTO was detected via Western blot. **(B, E)** Cell confluence (% of the surface area of cells) of FTO-upregulated AS and BE2 cells was dynamically monitored using IncuCyte ZOOM (*p < 0.05 and **p < 0.01). **(C)** Cell survival of FTO-upregulated AS and BE2 cells was detected using the CCK-8 array (*p < 0.05 and **p < 0.01). **(D)** Colony formation assays were performed in the AS and BE2 cells (*p < 0.05).

To explore the role of FTO in NB cells’ survival and proliferation, we designed an FTO overexpression plasmid (OE-FTO). This plasmid upregulated FTO expression (Figure 2A; Supplementary Datasheet 1). Two NB cell lines (AS and BE2) and NIH3T3 cells were transfected with an empty vector and the OE-FTO plasmid, and then cell confluence, survival, and proliferation were detected. The AS and BE2 cells’ confluence curves of the OE-FTO-transfected cells (red curve) were lower than those of the empty vector-transfected cells (blue curve) (Figure 2B). At 72 h after transfection, the cell confluence of the OE-FTO-transfected cells was 17.5% lower than that of empty vector-transfected cells in AS cells (*p* < 0.01) (Figure 2B), and 19.1% in BE2 cells (*p* < 0.05) (Figure 2B). However, in NIH3T3 cells, the confluence curves of OE-FTO and EV overlapped, showing no statistical significance in the 72 h endpoint analysis (Supplementary Datasheet 1). The results of the CCK-8 assay were consistent with the cell confluence data. Compared to the empty vector-transfected cells, the survival of OE-FTO-transfected cells decreased by 11.6% in AS cells (*p* < 0.05) (Figure 2C) and by 7.4% in BE2 cells (*p* < 0.01) (Figure 2C), and no statistically significant difference was observed in NIH3T3 cells ((*p* > 0.05) (Supplementary Datasheet 1). In the colony formation assay, the number of colonies formed by OE-FTO-transfected cells was 36.8% lower than that of empty vector-transfected cells in AS cells (*p* < 0.05) (Figure 2D), and 24.1% lower in BE2 cells (*p* < 0.05) (Figure 2D). Representative images of NB cells at 0 h and 72 h after transfection are shown in Figure 2E, indicating fewer cells in the OE-FTO transfection group than in the control group. These data indicate that the upregulation of FTO inhibits NB cells’ survival and proliferation.

### 3.3 Downregulation of FTO promoted NB cell proliferation

**FIGURE 3:**
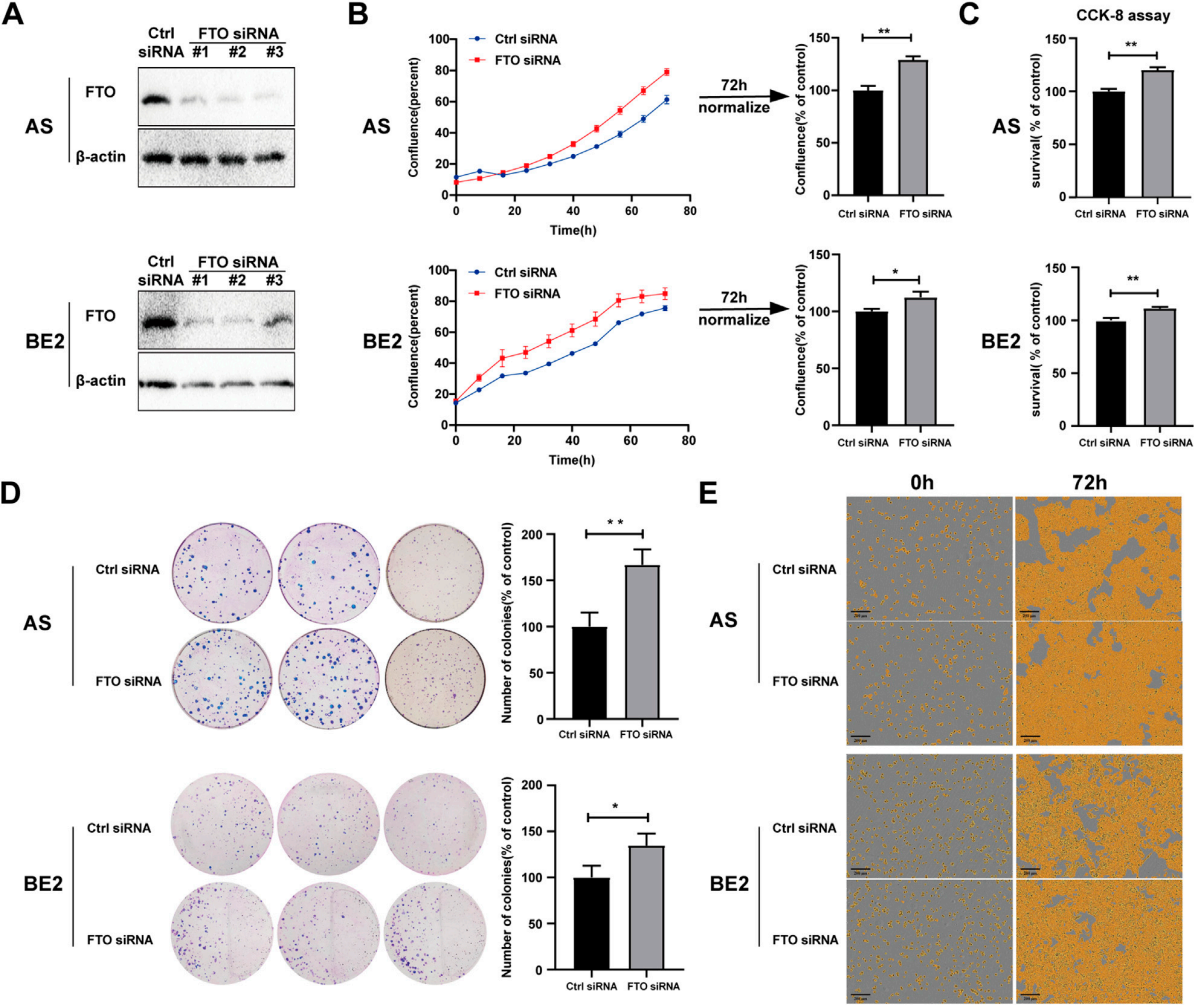
Downregulation of FTO promoted NB cell proliferation. **(A)** Expression of FTO in AS and BE2 cells transfected with FTO siRNAs detected through Western blot. **(B, E)** Cell confluence (% of the surface area of cells) of FTO-downregulated AS and BE2 cells was dynamically monitored using IncuCyte ZOOM (*p < 0.05 and **p < 0.01) **(C)** Cell survival of FTO-downregulated AS and BE2 cells was detected using the CCK-8 array (**p < 0.01) **(D)** Colony formation assays were performed in the AS and BE2 cells (*p < 0.05 and **p < 0.01).

To further explore the role of FTO in NB cells’ survival and proliferation, we designed three FTO siRNAs (#1, #2, and #3) that downregulated FTO expression (Figure 3A; Supplementary Datasheet 1). FTO siRNA#2 (FTO siRNA) was selected for subsequent studies because of its significant downregulating effects in both AS and BE2 cell lines. Two NB cell lines (AS and BE2) and NIH3T3 cells were transfected with control siRNA and FTO siRNA, and then cell confluence, survival, and proliferation were evaluated. AS and BE2 cells’ confluence curves of FTO siRNA-transfected cells (red curve) were higher than those of control siRNA-transfected cells (blue curve) (Figure 3B). At 72 h post-transfection, the confluence of the FTO siRNA-transfected cells was significantly increased by 29.0% compared to the control siRNA in AS cells (*p* < 0.01) (Figure 3B), and by 12.4% in BE2 cells (*p* < 0.05) (Figure 3B). However, in NIH3T3 cells, the confluence curves of FTO siRNA and control siRNA overlapped, showing no statistical significance at the 72 h endpoint analysis (Supplementary Datasheet 1). The results of the CCK-8 assay were consistent with the cell confluence data; the survival rates of the cells transfected with FTO siRNA showed a significant increase of 20.0% in AS cells (*p* < 0.01) (Figure 3C), an increase of 11.2% in BE2 cells (*p* < 0.01) (Figure 3C), and no statistically significant difference in NIH3T3 cells (*p* > 0.05) (Supplementary Datasheet 1) compared to the control siRNA. In the colony formation assay, the number of colonies formed by FTO siRNA-transfected cells was significantly increased by 66.8% in AS cells (*p* < 0.01) (Figure 3D) and by 34.7% in BE2 cells (*p* < 0.05) (Figure 3D), compared to the control siRNA. Representative images of NB cells at 0 h and 72 h after transfection are shown in Figure 3E, illustrating that there were higher number of cells in the FTO siRNA-transfected group than in the control siRNA-transfected group. These results suggest that downregulation of FTO significantly promotes NB cells’ survival and proliferation.

### 3.4 Effect of FTO on the sensitivity of NB cells to etoposide

Chemoresistance is the leading cause of treatment failure in patients with NB. To explore the effects of FTO on the sensitivity of NB cells to chemotherapeutic drugs, we selected etoposide, cisplatin, and paclitaxel—three compounds frequently used in the treatment of patients with NB.

**FIGURE 4:**
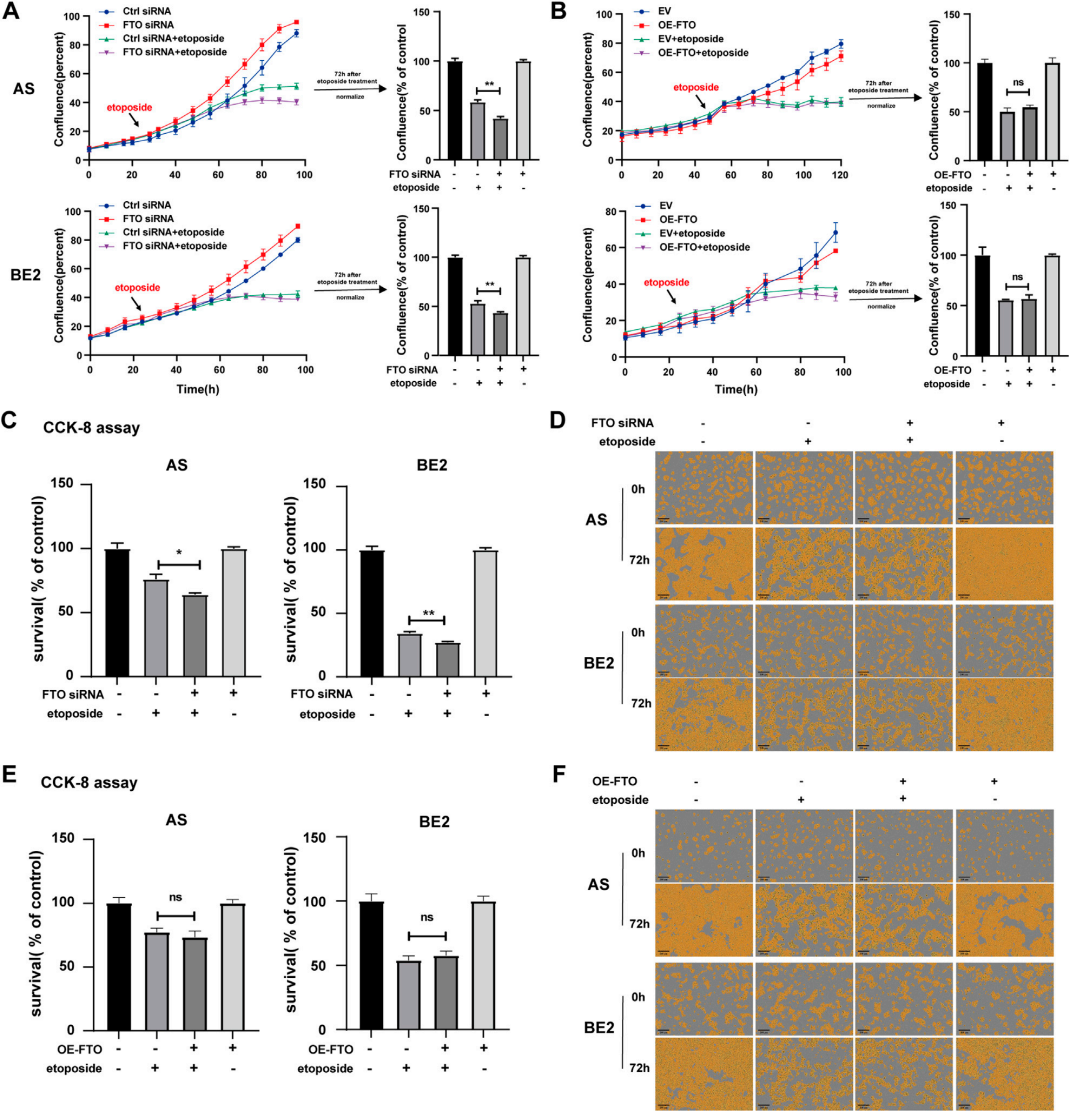
Effect of FTO on the sensitivity of NB cells to etoposide. **(A, D)** Cell confluence of AS and BE2 cells, transfected with FTO siRNAs followed by 72 h treatment of etoposide, was dynamically monitored using IncuCyte ZOOM and analyzed at the end of the experiment (**p < 0.01). **(B, F)** Cell confluence of AS and BE2 cells, transfected with OE-FTO followed by 72 h treatment of etoposide, was dynamically monitored using IncuCyte ZOOM and analyzed at the end of the experiment **(C)** Cell survival of AS and BE2 cells, transfected with FTO siRNAs followed by 72 h treatment of etoposide, was detected via the CCK-8 array (**p < 0.01). **(E)** Cell survival of AS and BE2 cells, transfected with OE-FTO followed by 72 h treatment of etoposide, was detected using the CCK-8 array.

To investigate the effect of FTO on the sensitivity of NB cells to etoposide, we transfected FTO siRNA or OE-FTO into AS and BE2 cells. The cells were then treated with etoposide for 72 h, and cell confluence and survival were assessed. After etoposide treatment, the confluence curves of FTO siRNA-transfected cells (purple curve) were lower than those of control siRNA-transfected cells (green curve) (Figure 4A, left). At 72 h post-etoposide treatment, the confluence of FTO siRNA-transfected cells was 16.3% lower than that of control siRNA-transfected cells in AS cells (*p* < 0.01) (Figure 4A, upper right), and 9.6% lower in BE2 cells (*p* < 0.01) (Figure 4A, lower right). The confluence of cells transfected with OE-FTO was not significantly different from that of cells transfected with the empty vector after the etoposide treatment (Figure 4B). The results of the CCK-8 assay were consistent with the cell confluence data; under the etoposide treatment, the cell survival of FTO siRNA-transfected cells was 11.9% lower than that of control siRNA-transfected cells in AS cells (*p* < 0.01), and 7.1% lower in BE2 cells (*p* < 0.01) (Figure 4C), but OE-FTO had no significant effect on the sensitivity of NB cells to etoposide (Figure 4E). Representative images of NB cells at 0 h and 72 h post-etoposide treatment are shown in Figures 4D, F, which corroborate the cell confluence and the CCK-8 assay. These findings suggest that downregulation of FTO enhances the sensitivity of NB cells to etoposide, whereas overexpression of FTO does not significantly affect cell survival following etoposide treatment.

### 3.5 Effect of FTO on the sensitivity of NB cells to cisplatin

**FIGURE 5:**
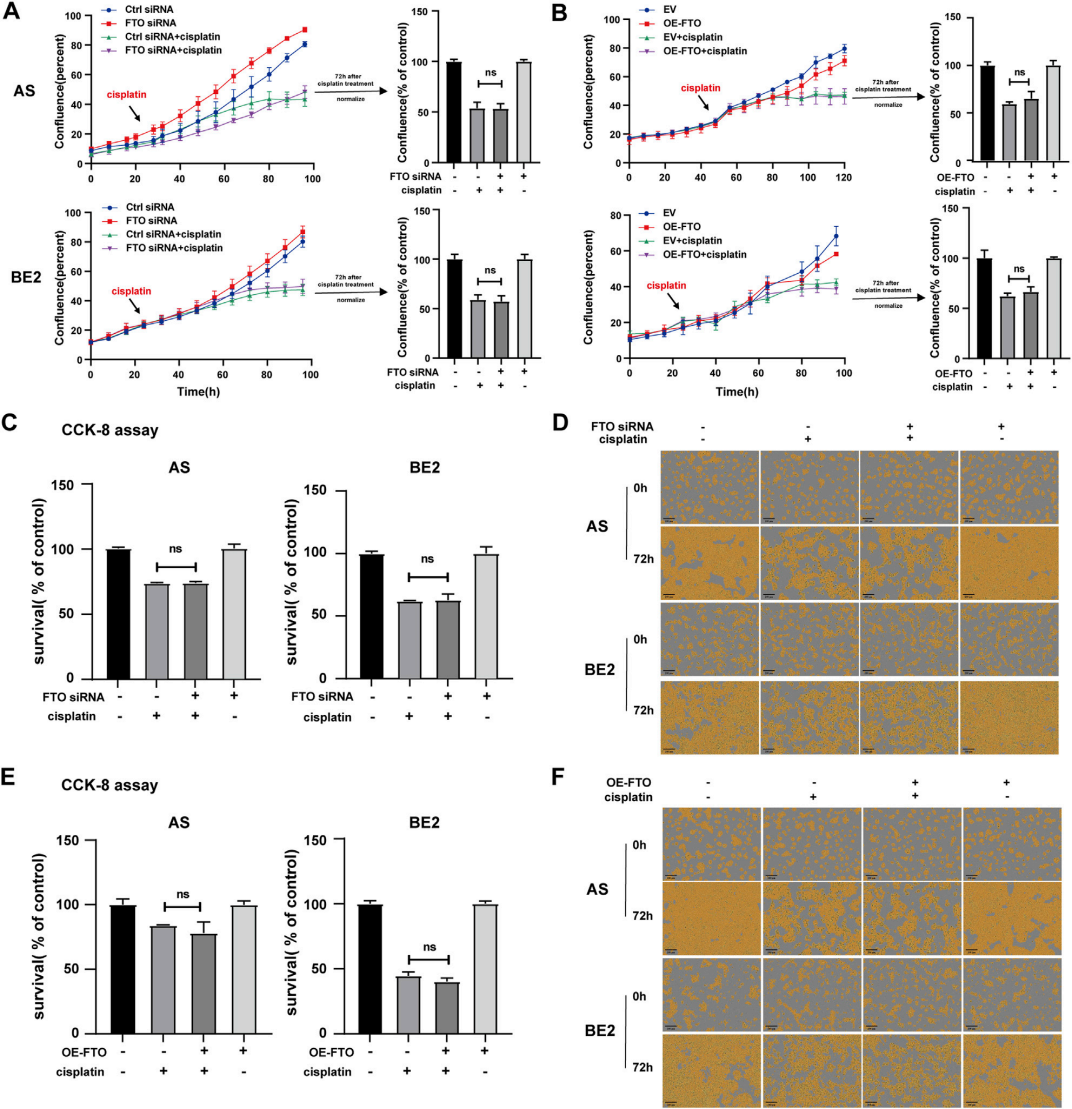
Effect of FTO on the sensitivity of NB cells to cisplatin. **(A, D)** Cell confluence of AS and BE2 cells, transfected with FTO siRNAs followed by 72 h treatment of cisplatin, was dynamically monitored using IncuCyte ZOOM and analyzed at the end of the experiment. **(B, F)** Cell confluence of AS and BE2 cells, transfected with OE-FTO followed by 72 h treatment of cisplatin, was dynamically monitored using IncuCyte ZOOM and analyzed at the end of the experiment. **(C)** Cell survival of AS and BE2 cells, transfected with FTO siRNAs followed by 72 h treatment of cisplatin, was detected *via* the CCK-8 array. **(E)** Cell survival of AS and BE2 cells, transfected with OE-FTO followed by 72 h treatment of cisplatin, was detected using the CCK-8 array.

To explore the effect of FTO on the sensitivity of NB cells to cisplatin, FTO siRNA or OE-FTO were transfected into AS cells and BE2. Subsequently, the cells were treated with cisplatin for 72 h, and cell confluence and survival rates were assessed. After cisplatin treatment, the confluence curves of FTO siRNA-transfected cells (purple curve) were slightly higher than those of control siRNA-transfected cells (green curve) (Figure 5A, left). Meanwhile, after cisplatin treatment, the confluence curves of cells transfected with OE-FTO (purple curve) were slightly lower than those of cells transfected with the empty vector (green curve) (Figure 5B, left). However, there were no significant differences in cell confluence 72 h after cisplatin treatment between cells transfected with FTO siRNA or OE-FTO compared to their respective controls (Figures 5A, B, right). These findings were consistent with the results of the CCK-8 assay, which showed no significant differences in cell survival rates between cells transfected with FTO siRNA or OE-FTO and their controls under cisplatin treatment (Figures 5C, E). Representative images of NB cells at 0 h and 72 h after cisplatin treatment are shown in Figures 5D, F, respectively, and similar trends were observed in the cell confluence and CCK-8 assay results. These findings suggest that FTO expression levels have no impact on the sensitivity of NB cells to cisplatin.

### 3.6 Effect of FTO on the sensitivity of NB cells to paclitaxel

**FIGURE 6:**
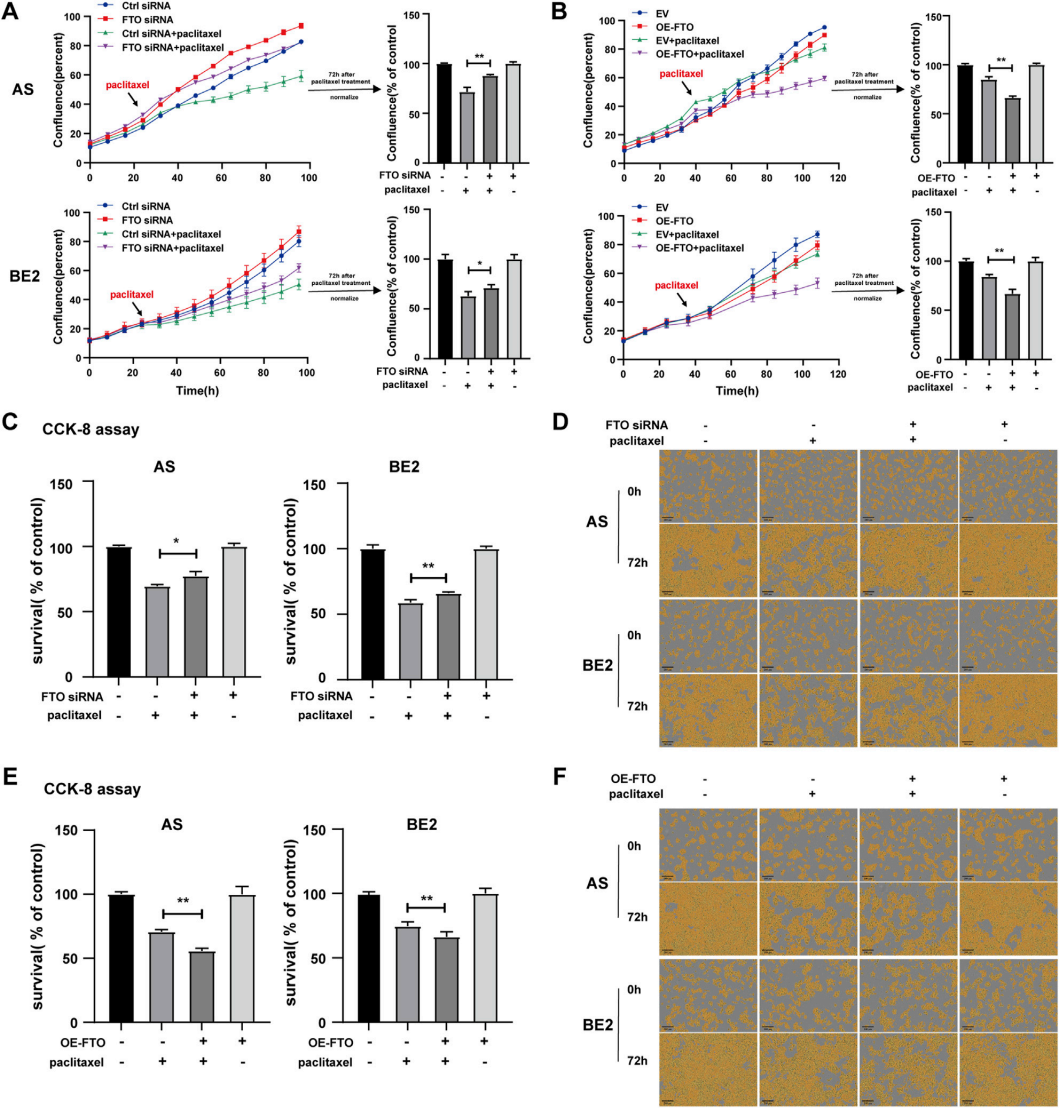
Effect of FTO on the sensitivity of NB cells to paclitaxel. **(A, D)** Cell confluence of AS and BE2 cells, transfected with FTO siRNAs followed by 72 h treatment of paclitaxel, was dynamically monitored using IncuCyte ZOOM and analyzed at the end of the experiment (*p < 0.05 and **p < 0.01). **(B, F)** Cell confluence of AS and BE2 cells, transfected with OE-FTO followed by 72 h treatment of paclitaxel, was dynamically monitored using IncuCyte ZOOM and analyzed at the end of the experiment (**p < 0.01). **(C)** Cell survival of AS and BE2 cells, transfected with FTO siRNAs followed by 72 h treatment of paclitaxel, was detected *via* the CCK-8 array (*p < 0.05 and **p < 0.01). **(E)** Cell survival of AS and BE2 cells, transfected with OE-FTO followed by 72 h treatment of paclitaxel, was detected using the CCK-8 array (**p < 0.01).

Furthermore, we investigated whether FTO affected the sensitivity of NB cells to paclitaxel. FTO siRNA or OE-FTO was transfected into AS and BE2 cells. Subsequently, the cells were treated with paclitaxel treatment for 72 h, after which the cell confluence and survival rates were detected. After paclitaxel treatment, the confluence curves of the cells transfected with FTO siRNA (purple curve) were significantly higher than those of cells transfected with control siRNA (green curve) (Figure 6A, left). Conversely, the confluence curves of the cells transfected with OE-FTO (purple curve) were significantly lower than those of cells transfected with the empty vector (green curve) after paclitaxel treatment (Figure 6B, left). At 72 h post-paclitaxel treatment, the confluence of the FTO siRNA-transfected cells was 16.1% higher than that of control siRNA-transfected cells in AS cells (*p* < 0.01) (Figure 6A, upper right), and 8.3% in BE2 cells (*p* < 0.05) (Figure 6A, lower right). Conversely, after paclitaxel treatment, OE-FTO-transfected cells exhibited significantly lower confluence than empty vector-transfected cells (for paclitaxel treatment, in AS cells, OE-FTO vs. empty vector: 66.3% and 85.1%, *p* < 0.01; in BE2 cells, OE-FTO vs. empty vector: 66.8% vs 84.3%, *p* < 0.01) (Figure 6B). The results of the CCK-8 assay were consistent with the cell confluence data; after paclitaxel treatment, the cell survival of the FTO siRNA-transfected cells was 7.9% higher than that of the control siRNA-transfected cells in AS cells (*p* < 0.05) (Figure 6C, left), and 7.1% in BE2 cells (*p* < 0.01) (Figure 6C, right). The survival rate of OE-FTO-transfected cells was significantly lower than that of empty vector-transfected cells (after paclitaxel treatment, in AS cells, OE-FTO vs. empty vector: 55.6% vs. 70.6%, *p* < 0.01; in BE2 cells, OE-FTO vs empty vector: 66.3% vs. 74.5%, *p* < 0.01) (Figure 6E). Representative images of NB cells at 0 h and 72 h after paclitaxel treatment are shown in Figures 6D, F, respectively, which corroborate the trends observed in cell confluence and CCK-8 assay results. These data collectively indicate that downregulation of FTO expression significantly reduces the sensitivity of NB cells to paclitaxel, whereas upregulation of FTO enhances the sensitivity of NB cells to paclitaxel.

### 3.7 Chemotherapeutic drugs and small-molecule inhibitors’ sensitivity analysis

**FIGURE 7:**
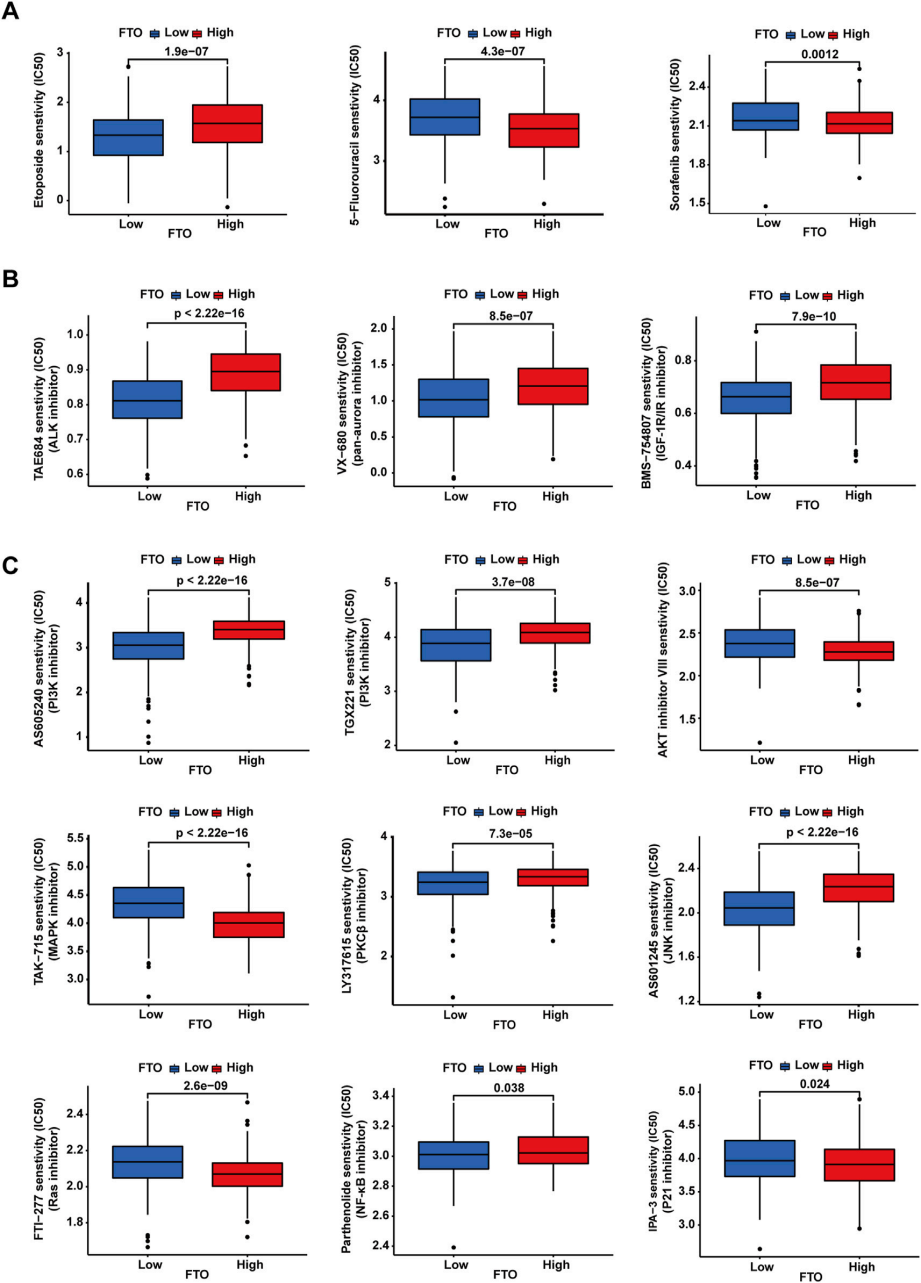
Chemotherapeutic drugs and small-molecule inhibitors’ sensitivity analysis. **(A–C)** Patients with low expression of FTO showed different sensitivities depending on the chemotherapeutic drugs or small-molecule inhibitors.

As FTO diversely influences the sensitivity of NB cells to etoposide, cisplatin, and paclitaxel, we explored the correlation between FTO expression and the sensitivity of patients with NB to different chemotherapeutic drugs and small-molecule inhibitors. We first downloaded a dataset from the GDSC that can be used for drug sensitivity analysis. We then imported this dataset into the “pRRophetic” package to train a predictive model for drug sensitivity. Subsequently, we applied this predictive model to the data of 498 patients with NB. The data were downloaded from the GEO database to evaluate the relationship between FTO gene expression levels and drug sensitivity. The results revealed distinct sensitivities based on FTO expression levels across different treatments (Figures 7A–C). Consistent with our results, low FTO expression was more sensitive to etoposide (Figure 7A). We did not obtain information about sensitivity to cisplatin and paclitaxel *via* the GDSC database, but low expression of FTO was less sensitive to 5-fluorouracil (an antimetabolite that inhibits thymidylate synthase) (Figure 7A), another commonly used chemotherapeutic drug, and sorafenib (Figure 7A), a multi-kinase inhibitor that is widely used in many types of cancer treatment. Anaplastic lymphoma kinase (ALK) and Aurora are two commonly mutated genes in NB, and low expression of FTO was more sensitive to TAE684 (ALK inhibitor), VX-680 (pan-Aurora inhibitor), and BMS-754807 (IGF-1R/IR inhibitor, which also inhibits Aurora) (Figure 7B). Furthermore, low expression of FTO exhibited different sensitivities to PI3K inhibitor, AKT inhibitor, MAPK inhibitor, PKCβ inhibitor, JNK inhibitor, Ras inhibitor, NF-κB inhibitor, and P21 inhibitor (Figure 7C).

## 4 Discussion

In this study, we investigated the correlation between FTO expression and the prognosis of patients with NB, its effect on NB cells survival and proliferation, and the sensitivity of NB cells to chemotherapeutic drugs (etoposide, cisplatin, and paclitaxel). Our findings indicate that high FTO expression was associated with a good prognosis in patients with NB. In addition, FTO overexpression inhibited cell proliferation, whereas FTO knockdown promoted NB cell proliferation in NB cells. Moreover, our study revealed that changes in FTO expression diversely influenced the sensitivity of NB cells to etoposide, cisplatin, and paclitaxel. Furthermore, we predicted the sensitivity of patients to various chemotherapeutic drugs and small-molecule inhibitors. This suggests that FTO expression levels have different effects on the sensitivity of NB cells to different chemotherapeutic drugs and small-molecule inhibitors, which may help guide clinicians in selecting the appropriate chemotherapeutic drugs or small-molecule inhibitors for treatments tailored to individual patient profiles.

Recent studies have highlighted the crucial role of FTO in tumorigenesis. Our results demonstrated a clear relationship between FTO expression and NB cell proliferation. Specifically, FTO downregulation promoted NB cell proliferation, whereas FTO upregulation inhibited cell proliferation. FTO plays a pivotal role in regulating NB cell division, potentially through its involvement in RNA demethylation and modulation of relevant signaling pathways. Huang et al. (2022) found that FTO knockdown significantly promoted glycolytic metabolism and proliferation of papillary thyroid cancer cells, whereas FTO overexpression inhibited glycolysis and proliferation of papillary thyroid cancer cells. Yang et al. (2019) reported that inhibition of FTO expression substantially suppressed melanoma growth, whereas FTO overexpression notably facilitated melanoma growth. Additionally, Zhou et al. (2021) reported that FTO overexpression promoted bladder cancer cell proliferation, whereas FTO downregulation inhibited bladder cancer cell proliferation. Collectively, these findings highlight the multifaceted role of FTO in the intricate landscape of cancer progression and its potential as a therapeutic target for various malignancies.

FTO is involved in chemotherapy resistance in various tumor types, including colorectal cancer (Lin et al., 2023a; Wang et al., 2022b), breast cancer (Ou et al., 2022; Wang et al., 2021), acute myeloid leukemia (Zhang et al., 2023), gastric cancer (Liu et al., 2023), and glioblastoma (Li et al., 2021). Notably, the effect of FTO on chemotherapy sensitivity in NB cells was highly specific to the type of chemotherapeutic agent used. Wang et al. (2021) found that FTO plays a role in STAT3-mediated doxorubicin resistance and compromises doxorubicin sensitivity in breast cancer cells. Lin et al. (2023a) reported that FTO can improve pancreatic cancer cell resistance to gemcitabine *via* the FTO-NEDD4-PTEN/PI3K/AKT axis by controlling the cell cycle, thereby influencing cell proliferation. A recent study reported that FTO-mediated LINC01134 stabilization promotes chemotherapy resistance to gemcitabine through the miR-140-3p/WNT5A/WNT pathway in pancreatic ductal adenocarcinoma (Lu et al., 2023). Liu et al. (2023) reported that FTO contributes to the increased resistance of gastric cancer cells to 5-fluorouracil (5-FU) by upregulating CDKAL1 and inducing mitochondrial fusion. Lin et al. (2023b) reported that the inhibition of FTO significantly reduces the tolerance of 5-FU in 5-FU-resistant colorectal cancer cells *via* the FTO-SIVA1 axis, whereas SIVA1 depletion can restore m6A-dependent 5-FU sensitivity in colorectal cancer cells. A recent study reported that bone marrow mesenchymal stem cells (BM-MSCs)-derived FTO-exosomes enhance cancer aggressiveness and cytosine arabinoside (Ara-C)-chemoresistance in acute myeloid leukemia by modulating the lncRNA GLCC1-IGF2BP1-c-Myc signaling pathway (Kou et al., 2023). FTO stabilizes long intergenic non-coding RNA for kinase activation (LINK-A) to promote cell proliferation and chemoresistance in esophageal squamous cell carcinoma (Nan et al., 2023). A recent study reported that the inhibition of FTO by regulating FOXO3 reduces the chemoresistance of acute myeloid leukemia cells through cell differentiation (Zhang et al., 2023). Wen et al. (2020) reported that downregulation of FTO protects bladder cancer cells from cisplatin-induced cytotoxicity. Zhang et al. (2022) reported that the knockdown of FTO reverses cisplatin resistance of cisplatin-resistant gastric cancer cells both *in vitro* and *in vivo* by inhibiting Unc-51-like kinase 1 (ULK1)-mediated autophagy. However, FTO exhibits completely different effects in response to various chemotherapeutic drugs in NB. FTO knockdown enhanced the sensitivity of NB cells to etoposide, whereas FTO overexpression did not affect its sensitivity. Both the downregulation and upregulation of FTO expression levels had no impact on the sensitivity of NB cells to cisplatin. Decreasing FTO expression levels reduced the sensitivity of NB cells to paclitaxel, whereas increasing FTO expression levels enhanced the sensitivity of NB cells to paclitaxel. In NB, the differential effects of FTO on cisplatin, etoposide, and paclitaxel can be attributed to their distinct mechanisms and how FTO interacts with the cellular pathways influenced by these drugs. Etoposide is a topoisomerase II inhibitor that induces DNA double-strand breaks. Reduced FTO expression may diminish cellular DNA repair capacity particularly double-strand break repair, thereby enhancing the sensitivity to etoposide. Cisplatin, an alkylating platinum-containing chemotherapeutic drug containing platinum, irreparably damages the DNA of dividing cells. Owing to the slight impact of changes in FTO expression levels on NB cell proliferation and considering cisplatin’s mechanism of DNA damage in dividing cells, we could potentially explain why FTO does not influence the sensitivity of NB cells to cisplatin. Paclitaxel, a taxane chemotherapeutic agent, disrupts microtubule structures that are essential for chromosomal movement during mitosis, thereby inhibiting cell division and leading to cell death. The effect of FTO on paclitaxel sensitivity suggests that it is involved in the cell cycle checkpoints. FTO may regulate proteins crucial for microtubule function and mitotic spindle formation, thereby affecting the response of cells to paclitaxel-induced mitotic arrest. FTO is a crucial factor that contributes to chemotherapy resistance in a variety of cancers; however, its mechanisms vary depending on the cancer type and specific molecular pathways involved. Understanding these mechanisms could potentially offer avenues for developing targeted therapies aimed at overcoming drug resistance in cancer treatment.

Our findings in the present study may provide guidance for the selection of appropriate treatment regimens. Patients with low FTO expression may be more sensitive to etoposide, ALK inhibitors, pan-Aurora inhibitors, IGF-1R/IR inhibitors, PI3K inhibitors, PKCβ inhibitors, JNK inhibitors and NF-κB inhibitors. Conversely, patients with high FTO expression may be more suitable for chemotherapeutic drugs, such as paclitaxel, 5-fluorouracil, and sorafenib, as well as small-molecule inhibitors such as AKT inhibitors, MAPK inhibitors, Ras inhibitors, and P21 inhibitors. Nevertheless, our study still had some limitations. Specifically, although our research indicates that FTO regulates the sensitivity of NB cells to etoposide and paclitaxel without altering their sensitivity to cisplatin, we have not elucidated the underlying mechanisms. Additionally, our study was confined to *in vitro* research on NB cells and has not been extended to *in vivo* models. We plan to focus on these issues in future studies. In conclusion, FTO overexpression inhibited cell proliferation, whereas FTO knockdown promoted NB cell proliferation. FTO has distinct effects on the sensitivity of NB cells to different chemotherapeutic drugs and small-molecule inhibitors.

## Data Availability

The original contributions presented in the study are included in the article/Supplementary Material; further inquiries can be directed to the corresponding authors.
