# Supplementary material for: FTO diversely influences sensitivity of neuroblastoma cells to various chemotherapeutic drugs
Source: Front Pharmacol. 2024 Sep 4;15:1384141. doi: 10.3389/fphar.2024.1384141 (PMC11409730; doi:10.3389/fphar.2024.1384141)
Supplement: Supplementary file 1 [file DataSheet1.pdf]

Supplementary Material

Supplementary Figures

Figure S1

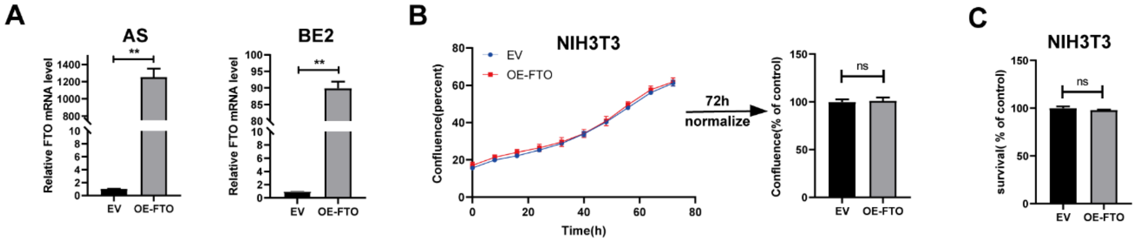

Supplementary Figure 1. (A) The expression of FTO on mRNA level in AS and BE2 cells transfected with FTO overexpression plasmids was detected by quantitative real-time PCR, OE-FTO vs. EV, ** $p < 0.01$. (B) The cell confluence (% of the surface area of cells) of NIH3T3 cells after overexpression was detected by IncuCyte Zoom. (C) The cell survival of FTO upregulation NIH3T3 cells was detected by the CCK8 assay, OE-FTO vs. EV, ns no significant difference.

Figure S2

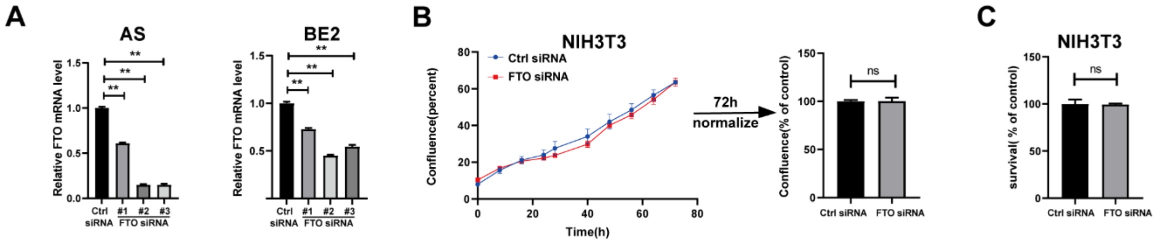

Supplementary Figure 2. (A) The mRNA expression of FTO on mRNA level in AS and BE2 cells transfected with FTO siRNAs was detected by quantitative real-time PCR, FTO siRNA vs. ctrl siRNA, ** $p < 0.01$. (B) The cell confluence (% of the surface area of cells) of NIH3T3 cells after FTO downregulated was detected by IncuCyte Zoom. (C) The cell survival of FTO downregulation NIH3T3 cells was detected by the CCK8 assay, FTO siRNA vs. ctrl siRNA, ns no significant difference.
